# How leader greed relates to employee unethical behavior: a moderated mediation model of leader self-serving behavior and employee moral attentiveness

**DOI:** 10.3389/fpsyg.2026.1929238

**Published:** 2026-09-18

**Authors:** Wenjing Gui, Xin Luo, Fang-Hsuan Hsueh, Xinyue Yuan

**Affiliations:** 1 School of Journalism, Communication University of China, Beijing, China; 2 Chongqing Aerospace Polytechnic, Chongqing, China; 3 School of Public Administration, Hohai University, Nanjing, China

**Keywords:** employee moral attentiveness, employee unethical behavior, leader greed, leader self-serving behavior, social cognitive theory, trickle-down effect

## Abstract

**Introduction:**

Leader greed has potentially important implications for employee behavior, yet its relationship with employee unethical behavior and the underlying mechanisms remain unclear. This study investigates how leader greed is associated with employee unethical behavior by examining the mediating role of leader self-serving behavior and the moderating role of employee moral attentiveness.

**Methods:**

We conducted two three-wave, time-lagged studies to test our hypotheses. Study 1 used multisource data from 231 employees nested within 62 leaders to examine the mediating role of leader self-serving behavior in the relationship between leader greed and employee unethical behavior. Study 2 (*N* = 401) replicated these findings and further tested the moderated mediation model, focusing on the moderating role of employee moral attentiveness.

**Results:**

Leader greed was positively related to employee unethical behavior, and this relationship was mediated by leader self-serving behavior. Furthermore, employee moral attentiveness attenuated the positive relationship between leader self-serving behavior and employee unethical behavior, as well as the indirect effect of leader greed on employee unethical behavior through leader self-serving behavior.

**Discussion:**

These findings extend greed research from an intrapersonal focus to an interpersonal and trickle-down perspective, revealing how leader greed relates to employee unethical behavior through leader self-serving behavior. By identifying employee moral attentiveness as an important boundary condition, our findings offer theoretical and practical insights into when and how leader greed is linked to interpersonal processes and employee ethical conduct within organizations.

## 1 Introduction

Greed is defined as “the tendency to always want more and never being satisfied with what one currently has” (Seuntjens et al., 2015, p. 917). As one of the oldest and most controversial social constructs in human history, greed has consistently been a focal point of discussion across multiple disciplines (Haynes et al., 2017; Takacs-Haynes et al., 2026; Wang and Murnighan, 2011). In organizational contexts, leader greed may pose particular risks because leaders not only control the allocation of organizational resources (Rus et al., 2010a) but also play a central role in defining behavioral norms within organizations (Paterson and Huang, 2019). At the macro level, CEO greed has been associated with lower corporate social responsibility and longer recovery times following systemic shocks (Sajko et al., 2021). At the micro level, leader greed may elicit negative emotional reactions and dysfunctional behavioral responses among employees (Carnevale et al., 2021).

Despite growing academic attention to greed, current research still exhibits two major limitations. First, most studies treat greed as a dispositional trait (Krekels and Pandelaere, 2015; Seuntjens et al., 2015; Zeelenberg and Breugelmans, 2022) and focus primarily on greedy individuals’ own behaviors and outcomes, such as lower levels of prosocial behavior (Bao et al., 2022), greater gambling participation (Weller et al., 2026), and lower life satisfaction (Crusius et al., 2021). However, greed possesses an inherent social nature with profound interpersonal implications (Seuntjens et al., 2015; Takacs-Haynes et al., 2026; Wang and Murnighan, 2011). Although recent research has begun to examine the interpersonal implications of leader greed (Carnevale et al., 2021; Zhou et al., 2026), both its relationship with employee unethical behavior and the processes underlying this relationship remain underexplored. Second, existing literature largely focuses on the direct relationship between greed and negative outcomes. It is therefore important to move beyond asking whether greed is harmful and examine how and when greed relates to negative outcomes (Takacs-Haynes et al., 2026; Wang and Murnighan, 2011). More specifically, in organizational contexts, this association between leader greed and employee unethical behavior may involve specific behavioral pathways (e.g., leader self-serving behavior) and vary with individual differences (e.g., employee moral attentiveness).

Grounded in social cognitive theory (Bandura, 1986, 1991) and the trickle-down model (Mayer et al., 2009), this study proposes a moderated mediation model to examine the mechanisms linking leader greed to employee unethical behavior. Social cognitive theory posits that individual behavior results from dynamic, triadic reciprocal interactions among the environment, individual differences, and behavior (Bandura, 1986, 1991). Within this framework, leader behavior constitutes an important environmental signal in organizations, conveying information about ethical norms and social expectations (Bandura, 1986; Mayer et al., 2009), while individual differences in moral cognition provide a basis for interpreting and processing these signals (Reynolds, 2008). Specifically, leaders’ dispositional greed may be expressed in self-serving behavior in the workplace (Haynes et al., 2015; Wang and Murnighan, 2011), and such conduct may then provide employees with cues about what is accepted or expected (Mayer et al., 2009). Through observational learning, employees may interpret these cues as indicating that prioritizing personal interests is acceptable (Bandura, 1986; Mayer et al., 2009). When personal interests subsequently conflict with ethical standards, this interpretation may facilitate moral disengagement and make unethical behavior more likely to occur (Bandura, 1999; Moore et al., 2012). However, employees are not passive recipients of environmental signals. Instead, they filter and interpret external cues based on their moral agency (Bandura, 1991). To this end, we introduce employee moral attentiveness (Reynolds, 2008) as a moderating variable. Individuals high in moral attentiveness are more attuned to moral cues and tend to interpret environmental information through a moral lens (Reynolds, 2008), making them more likely to attend to the potential ethical implications of leader self-serving behavior and less likely to uncritically imitate it. In contrast, individuals low in moral attentiveness are less likely to interpret leader self-serving behavior through a moral lens or consider its ethical implications (Reynolds, 2008; van Gils et al., 2015), which may facilitate moral disengagement and increase the likelihood of unethical behavior (Bandura, 1999; Moore et al., 2012).

By constructing this moderated mediation model, the present study aims to make the following contributions. First, by extending greed research beyond its predominantly intrapersonal focus to examine the interpersonal influences of greed, this study enriches our understanding of the relationship between leader greed and employee unethical behavior. In doing so, we build on Wang and Murnighan’s (2011) view that greed contributes to important personal and interpersonal dynamics and address the relative scarcity of empirical work examining such dynamics. Second, by integrating social cognitive theory (Bandura, 1986, 1991) with the trickle-down model (Mayer et al., 2009), we identify leader self-serving behavior as a mediating pathway and clarify the behavioral mechanism underlying the trickle-down effect of leader greed. Third, we identify employee moral attentiveness (Reynolds, 2008) as an important boundary condition that helps explain why employees may respond differently to leader greed. Together, these contributions extend research on the dark side of leadership and employee ethical behavior by offering a leader-greed perspective that highlights the insatiable desire to acquire more that characterizes greed (Krekels and Pandelaere, 2015; Seuntjens et al., 2015).

## 2 Theoretical background and hypothesis development

### 2.1 Leader greed and leader self-serving behavior

Leader greed is a relatively stable dispositional tendency to always want more and never be satisfied with what one currently has (Seuntjens et al., 2015). By contrast, leader self-serving behavior refers to actions through which leaders prioritize their own interests and well-being over followers’ needs and organizational goals (Camps et al., 2012). Thus, the former captures an underlying desire for more (Krekels and Pandelaere, 2015; Seuntjens et al., 2015), whereas the latter reflects actions taken to advance leaders’ own interests (Camps et al., 2012). In organizational contexts, leader self-serving behavior may represent one way in which leader greed is expressed.

According to social cognitive theory (Bandura, 1986, 1991), a dynamic interplay exists among an individual’s internal traits, external environment, and behavioral manifestations. In organizational contexts, leaders typically possess authority and control over important resources (Keltner et al., 2003; Rus et al., 2010a), and these environmental conditions can shape how their internal tendencies are expressed in behavior (Haynes et al., 2015). Power may strengthen the influence of leaders’ internal tendencies on their behavior. When making decisions, leaders with greater power tend to rely less on external information and more on their internal states (Rus et al., 2010a; DeCelles et al., 2012), including greed. Since greed is an internal disposition, power may give leaders greater latitude to act on it. Moreover, research on senior leaders suggests that position-related discretion and power may provide opportunities to pursue additional material gains (Haynes et al., 2017). Self-serving behavior may thus provide a means for leaders to pursue personal gain (Camps et al., 2012). Given their continuing desire for more (Seuntjens et al., 2015), leaders high in greed may be more inclined to act in self-serving ways (Haynes et al., 2015; Wang and Murnighan, 2011), such as claiming undue credit for group accomplishments, allocating shared resources in their own favor, or shirking their own responsibilities (Rus et al., 2010a,b). Thus, leaders’ power and access to opportunities may enable them to express their greed through self-serving behavior. Accordingly, we propose:

*H1*: Leader greed is positively associated with leader self-serving behavior.

### 2.2 The mediating role of leader self-serving behavior

The trickle-down model proposes that leaders’ behaviors and ethical standards can be transmitted downward through organizational hierarchies (Mayer et al., 2009). Social cognitive theory further suggests that employees learn by observing role models (Bandura, 1986), and leaders’ conduct can therefore signal which behaviors are accepted or expected in the organization (Mayer et al., 2009). Since leader greed is an internal disposition (Seuntjens et al., 2015), it is less readily observable to subordinates than leaders’ overt conduct. In organizational settings, dispositional tendencies can manifest in work behavior (Tett and Burnett, 2003). Such behavior is more readily observable to subordinates and can therefore serve as a basis for observational learning (Bandura, 1986). Leader self-serving behavior may therefore represent one observable way in which leader greed is expressed and provide a behavioral link between leader greed and employee unethical behavior. We develop this argument by considering employees’ moral self-regulation and normative learning within teams.

On the one hand, a self-regulatory perspective suggests that observing leader self-serving behavior may affect how employees morally evaluate their own conduct. Social learning involves not only imitating a model’s specific actions but also learning the broader principles they reflect, which may guide conduct in situations beyond those directly observed (Bandura, 1971; Saher et al., 2024). In the present context, subordinates who observe a leader pursuing personal gain through self-serving behavior may infer that prioritizing one’s own interests is acceptable. When employees subsequently face a conflict between personal gain and ethical standards, this learned principle may facilitate moral disengagement by providing a justification for placing self-interest first (Bandura, 1991, 1999; Lian et al., 2022; Uppal and Bansal, 2023). By reframing self-interested conduct as acceptable, employees may selectively disengage the moral self-sanctions that would otherwise restrain such conduct (Bandura, 1999). They may consequently experience less of the guilt and shame normally associated with unethical conduct (Bandura, 1999; Bandura et al., 1996; Mesdaghinia et al., 2023). With fewer internal constraints on their conduct, employees may become more likely to engage in workplace unethical behavior (Bandura, 1999; Moore et al., 2012; Uppal and Bansal, 2023).

On the other hand, a normative learning perspective suggests that leader self-serving behavior may change how employees interpret and respond to the pursuit of personal interests in their team. As central and visible figures within the team (Haynes et al., 2015; Takacs-Haynes et al., 2026), leaders provide team members with a common behavioral referent and convey cues about which behaviors are accepted or expected (Mayer et al., 2009). When leader self-serving behavior appears to be tolerated or goes unpunished, team members may infer through vicarious learning that similar behavior is acceptable (Bandura, 1986). As team members observe both the leader’s behavior and one another’s responses, their perceptions of acceptable behavior may gradually converge and be reinforced through ongoing interactions, thereby leading them to view self-serving behavior as increasingly acceptable within the team (Robinson and O’Leary-Kelly, 1998; Mayer et al., 2009; Takacs-Haynes et al., 2026). In turn, employees may become more willing to protect their interests at the expense of ethical standards, particularly when competing for resources. Over time, this growing acceptance of self-serving behavior may influence which values employees consider important and how they evaluate ethical conduct (Takacs-Haynes et al., 2026) and make unethical behavior more likely to spread among team members (Ashforth and Anand, 2003; Mayer et al., 2009).

In sum, leader self-serving behavior may serve as a mediating mechanism linking leader greed to employee unethical behavior through a trickle-down effect. Accordingly, we propose:

*H2*: Leader self-serving behavior is positively associated with employee unethical behavior.

*H3*: Leader self-serving behavior mediates the relationship between leader greed and employee unethical behavior.

### 2.3 The moderating role of employee moral attentiveness

Social cognitive theory posits that individuals possess self-regulatory capabilities that shape how they process and respond to environmental information (Bandura, 1986). One important individual difference relevant to this process is moral attentiveness, defined as “the extent to which an individual chronically perceives and considers morality and moral elements in his or her experiences” (Reynolds, 2008, p. 1027). Employees high in moral attentiveness are more likely to identify potential moral cues in the work environment and incorporate them into their decision-making processes. As a chronically accessible framework for interpreting moral issues (Reynolds, 2008), moral attentiveness may also buffer the relationships between work-related factors and employee unethical behavior (Murtaza et al., 2026; Ouyang et al., 2022).

Specifically, we focus on employee moral attentiveness as a moderator of the second stage of the mediation process (i.e., the path linking leader self-serving behavior to employee unethical behavior). Prior research suggests that individuals high in moral attentiveness are more likely to base their responses on the moral cues they observe in a situation (Reynolds, 2008; van Gils et al., 2015). Whereas leader greed is an internal disposition (Seuntjens et al., 2015) that may not be directly observable to employees, leader self-serving behavior provides an overt and concrete cue. Employee moral attentiveness should therefore be particularly relevant to how employees attend to and interpret a leader’s self-serving behavior.

For employees high in moral attentiveness, this attention to moral cues acts as a perceptual lens through which ambiguous environmental signals are transformed into salient moral judgments (Reynolds, 2008). When confronted with a leader’s self-serving behavior, these employees are more likely to recognize its potential ethical implications and evaluate the behavior through a moral frame (Reynolds, 2008; van Gils et al., 2015). This cognitive filtering may attenuate the positive relationship between leader self-serving behavior and employee unethical behavior. Conversely, employees low in moral attentiveness are less sensitive to potential moral cues in the environment and may be more likely to construe such situations as amoral, that is, as not requiring moral judgment (Tenbrunsel and Smith-Crowe, 2008; van Gils et al., 2015). Under these circumstances, they may interpret leader self-serving behavior through an instrumental frame (Desmet et al., 2015), focusing more on its potential utility than on its ethical implications. When personal interests conflict with ethical standards, this instrumental interpretation may facilitate the selective disengagement of moral self-sanctions and make unethical behavior more likely (Bandura, 1999; Moore et al., 2012).

Taken together, employee moral attentiveness may serve as a critical boundary condition that not only moderates employees’ reactions to leader self-serving behavior but also weakens the indirect relationship between leader greed and employee unethical behavior through leader self-serving behavior. Accordingly, we propose:

*H4*: Employee moral attentiveness moderates the relationship between leader self-serving behavior and employee unethical behavior, such that the relationship is weaker when employee moral attentiveness is high, and stronger when employee moral attentiveness is low.

*H5*: Employee moral attentiveness moderates the indirect effect of leader greed on employee unethical behavior through leader self-serving behavior, such that the indirect effect is weaker when employee moral attentiveness is high and stronger when employee moral attentiveness is low.

The proposed theoretical model is shown in Figure 1.

**FIGURE 1:**
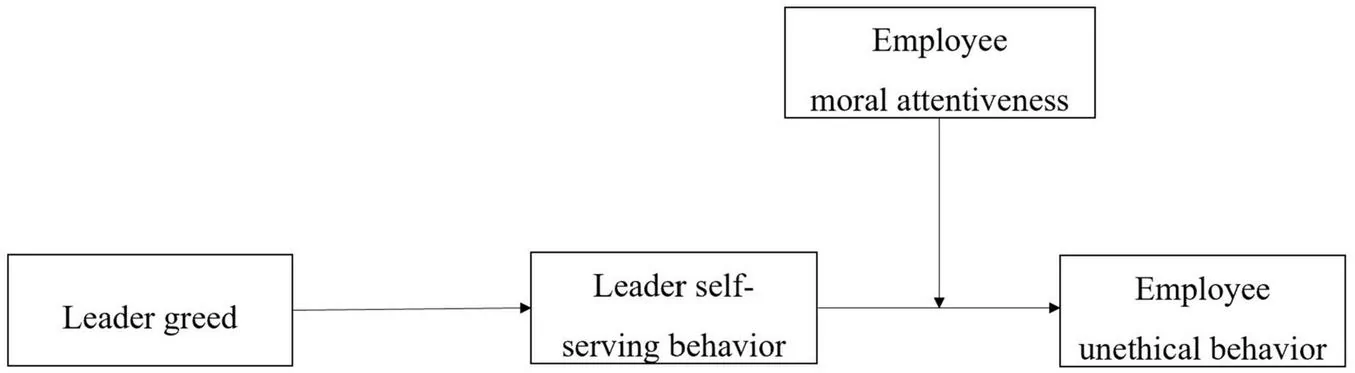
The theoretical model.

## 3 Study 1

### 3.1 Methods

#### 3.1.1 Sample and procedure

To test our hypotheses, we employed a three-wave, time-lagged field survey design to reduce common method bias (Podsakoff et al., 2003) and to temporally separate the measurement of our focal variables in accordance with the hypothesized sequence. Collaborating with human resources departments, we recruited leaders and their direct reports from several organizations to participate. Before data collection, participants were assured that all responses would be kept strictly confidential and used only for research purposes; participation was voluntary, and no identifying information would be disclosed to their organizations. To track respondents across the three waves and match leader-follower dyads, we utilized unique identification codes assigned at Wave 1.

Data were collected in three waves, with a 4-week interval between each wave. At Time 1 (T1), leaders completed a self-report measure of dispositional greed. At Time 2 (T2), employees rated their direct leader’s self-serving behavior. At Time 3 (T3), leaders rated each of their direct reports’ unethical behavior at work. Employee-level and leader-level demographic variables (i.e., age, gender, education, work experience, and organizational tenure) were collected at T1 as controls.

After matching responses across the three waves and dropping incomplete cases, the final sample consisted of 231 employees nested within 62 leaders (average team size = 3.73). In the employee sample, the mean age category was 2.93 (on a 1–4 scale) and 39.8% were female. In the leader sample, the mean age category was 3.30 and 19.5% were female.

#### 3.1.2 Measures

Following Brislin’s (1980) back-translation procedure, all items were translated from English into Chinese by two bilingual researchers and back-translated by two independent researchers. Discrepancies were resolved through discussion. Unless otherwise noted, responses were provided on a five-point Likert-type scale (1 = strongly disagree/never; 5 = strongly agree/always).

##### 3.1.2.1 Leader greed (T1, self-rated by leaders)

We measured leader greed with a 7-item scale (Seuntjens et al., 2015) that captures the leader’s general trait-like tendency to desire more than what they currently have. Since each leader provided a single score, this variable has no within-leader variance and is treated as a pure Level-2 (leader-level) construct. Cronbach’s α = 0.85; McDonald’s ω = 0.85.

##### 3.1.2.2 Leader self-serving behavior (T2, rated by employees)

Employees rated their leader’s self-serving behavior (LSSB; Rus et al., 2010a) on an 8-item scale tapping the extent to which the leader places personal benefit above team or organizational interests. Cronbach’s α = 0.78; McDonald’s ω = 0.81.

##### 3.1.2.3 Employee unethical behavior (T3, rated by leaders)

Leaders rated each of their direct reports on a 5-item unethical-behavior scale (Paterson and Huang, 2019) adapted from prior research (e.g., Bennett and Robinson, 2000). Because the same leader rated multiple subordinates, we modeled employee unethical behavior as a Level-1 outcome nested within leaders. Cronbach’s α = 0.66; McDonald’s ω = 0.65.

##### 3.1.2.4 Control variables

At the individual (Level-1) level, we controlled for employees’ age, gender, education, work experience, organizational tenure, and time with the current leader. At the team (Level-2) level, we controlled for the leader’s age, gender, education, work experience, and tenure. These controls follow recommended practice for control variable inclusion (Bernerth and Aguinis, 2016) and parallel the demographic controls used in recent leadership research (e.g., Zhang et al., 2024).

#### 3.1.3 Analytical strategy

Because our data have a nested structure (employees nested within leaders) and our theoretical model involves a Level-2 independent variable (leader greed), a mediator assessed by employees with substantial between-leader variance, and a Level-1 dependent variable (employee unethical behavior), we tested the hypotheses using a 2-1-1 multilevel mediation model (Preacher et al., 2010). This specification is analytically equivalent to the multilevel path model used in recent time-lagged mediation studies in leadership research (e.g., Zhang et al., 2024).

Following Zhang et al. (2024), we decomposed leader self-serving behavior into between-leader (LSSB_between, leader mean) and within-leader (LSSB_within = raw score – leader mean; i.e., group-mean centered) components so that the Level-2 mediating path from leader greed through LSSB_between to employee unethical behavior could be estimated without contamination by Level-1 variance. Leader greed and Level-2 control variables were grand-mean centered; Level-1 controls were left uncentered to preserve their raw interpretation. The mediator equation was estimated via ordinary least squares (OLS) at Level 2 (*N* = 62 leaders); the outcome equation was estimated via multilevel modeling (MLM) with a random intercept at the leader level using maximum likelihood. Explicitly, Hypothesis 1 is tested by the Level-2 path *a* from leader greed to LSSB_between in the mediator equation; Hypothesis 2 is tested by the between-leader path *b* from LSSB_between to employee unethical behavior in the outcome equation; and Hypothesis 3 is tested by the Monte Carlo confidence interval for the Level-2 indirect effect *a* × *b*.

To obtain an accurate test of the Level-2 indirect effect (path *a* × path *b*), we implemented the Monte Carlo method for assessing mediation with 20,000 replications following Selig and Preacher (2008) and Preacher et al. (2010). Because Monte Carlo resampling directly simulates the product distribution of *a* × *b* from the asymptotic sampling distributions of the two path coefficients, it yields accurate confidence intervals even when the product is non-normal and when the between-leader sample is modest.

We adopted the two-step approach (OLS mediator equation at Level 2 and random-intercept MLM for the outcome, with Monte Carlo integration of the indirect effect; Preacher et al., 2010; Selig and Preacher, 2008) for a substantive reason: our Level-2 sample contains only 62 leaders with a median team size of 3–4. Simulation research suggests that doubly latent multilevel structural equation modeling (MSEM) requires substantially larger numbers of clusters to yield stable estimates, and that, when the number of clusters is small, its latent group-mean corrections may reduce rather than improve estimation accuracy (Lüdtke et al., 2011; McNeish and Stapleton, 2016).

### 3.2 Results

#### 3.2.1 Descriptive analysis

##### 3.2.1.1 Data aggregation

Because leader self-serving behavior was rated by multiple employees about the same leader, we examined whether aggregating employee responses to the leader level was justified. We computed the intraclass correlation coefficients ICC(1) and ICC(2), and the within-group agreement index *r*_wg(J) (James et al., 1984). For leader self-serving behavior, ICC(1) = 0.92, ICC(2) = 0.98, *F*(61, 169) = 46.47, *p* < 0.001; the median *r*_wg(J) = 0.96, with 98.2% of teams exhibiting *r*_wg(J) ≥ 0.70. These statistics strongly support aggregating individual responses to the leader level (Bliese, 2000; LeBreton and Senter, 2008). For employee unethical behavior, ICC(1) = 0.43, ICC(2) = 0.74, *F*(61, 169) = 3.86, *p* < 0.001, confirming meaningful between-leader variance and justifying our multilevel modeling approach.

##### 3.2.1.2 Null model

Consistent with multilevel analytic practice (Bliese, 2000; Zhang et al., 2024), we conducted null-model variance decomposition for employee unethical behavior. Results revealed substantial variance at both levels: 44% between-leader (τ00 = 0.138) and 56% within-leader (σ^2^ = 0.173), further supporting the multilevel modeling approach.

##### 3.2.1.3 Descriptive statistics and correlations

Table 1 presents means, standard deviations, Cronbach’s alphas, and within- and between-level correlations among the focal variables. At the between-leader level, leader greed was positively correlated with leader self-serving behavior (*r* = 0.32, *p* = 0.011), providing initial support for Hypothesis 1. Leader self-serving behavior and employee unethical behavior were positively correlated at the between-leader level (*r* = 0.17, *p* = 0.19), in the expected direction.

**TABLE 1:** Means, standard deviations, and correlations among core study variables (study 1).

Variables	M	SD _(within)_	SD_ (between)_	1	2	3
1. Leader greed (T1)	2.94	0.00	0.79	(0.85)	0.32*	−0.08
2. Leader self-serving behavior (T2)	1.63	0.14	0.63	0.00	(0.78)	0.17
3. Employee unethical behavior (T3)	2.11	0.35	0.48	−0.00	0.01	(0.66)

*N* = 231 employees nested within 62 leaders. Correlations below the diagonal are within-level correlations; correlations above the diagonal are between-level correlations. Cronbach’s alphas are in parentheses on the diagonal. T1 = Time 1 (leader self-report); T2 = Time 2 (employee rating of leader); T3 = Time 3 (leader rating of employee). Because leader greed was measured at the leader level, no within-level correlation is computed.

*p < 0.05.

#### 3.2.2 Confirmatory factor analyses

Following common practice in organizational research (e.g., Landis et al., 2000; Little et al., 2002; Zhang et al., 2024), we created three item parcels for each latent construct and conducted a series of confirmatory factor analyses (CFA) to examine the discriminant validity among leader greed, leader self-serving behavior, and employee unethical behavior.

As reported in Table 2, the hypothesized three-factor model [χ^2^(24) = 85.43, χ^2^/df = 3.56, comparative fit index (CFI) = 0.92, Tucker–Lewis index (TLI) = 0.88, root-mean-square error of approximation (RMSEA) = 0.105] fit significantly better than all plausible alternative models: (a) a two-factor model combining leader greed and leader self-serving behavior into one factor [χ^2^(26) = 270.15, Δχ^2^(2) = 184.72, *p* < 0.001]; (b) a two-factor model combining leader self-serving behavior and employee unethical behavior [χ^2^(26) = 232.12, Δχ^2^(2) = 146.69, *p* < 0.001]; (c) a two-factor model combining leader greed and employee unethical behavior [χ^2^(26) = 271.86, Δχ^2^(2) = 186.43, *p* < 0.001]; and (d) a one-factor model in which all items loaded on a single latent variable [χ^2^(27) = 441.36, Δχ^2^(3) = 355.93, *p* < 0.001]. Although TLI and RMSEA fell short of conventional benchmarks, RMSEA is known to be inflated in models with small degrees of freedom and modest sample sizes (Kenny et al., 2015); our parceled model has only 24 degrees of freedom with *N* = 231. Most importantly, the CFA was conducted to assess discriminant validity, and the three-factor model outperformed every alternative by a wide margin. These results, together with our time-lagged, multi-source design (leader self-reports at T1; employee ratings at T2; leader ratings of employees at T3), suggest that common method variance (CMV) is unlikely to be a substantial concern in Study 1.

**TABLE 2:** Results of confirmatory factor analyses (Study 1).

Model	χ ^2^	df	χ ^2^/df	CFI	TLI	RMSEA	Δχ ^2^
Three-factor model (hypothesized)	85.43	24	3.56	0.918	0.877	0.105	
Two-factor: LG + LSSB combined	270.15	26	10.39	0.674	0.549	0.202	184.72***
Two-factor: LSSB + EUB combined	232.12	26	8.93	0.725	0.619	0.186	146.69***
Two-factor: LG + EUB combined	271.86	26	10.46	0.672	0.545	0.203	186.43***
One-factor model	441.36	27	16.35	0.447	0.262	0.258	355.93***

*N* = 231. LG, leader greed; LSSB, leader self-serving behavior; EUB, employee unethical behavior.

***p < 0.001.

#### 3.2.3 Hypothesis testing

Hypothesis 1 predicted that leader greed would be positively related to leader self-serving behavior. Results from the Level-2 mediator equation show that, after controlling for leader demographics, leader greed was significantly positively associated with leader self-serving behavior (*b* = 0.30, *SE* = 0.10, *p* = 0.004). Hypothesis 1 was supported.

Hypothesis 2 predicted that leader self-serving behavior would be positively related to employee unethical behavior. In the outcome equation, after partialling out leader greed, Level-1 controls, and Level-2 controls, the between-leader component of leader self-serving behavior was significantly positively related to employee unethical behavior (*b* = 0.21, *SE* = 0.10, *p* = 0.032). The within-leader component of self-serving behavior did not significantly predict employee unethical behavior (*b* = −0.01, *SE* = 0.20, *p* = 0.951), which is consistent with our theoretical framing that self-serving behavior is fundamentally a leader-level construct that shapes the shared ethical climate experienced by subordinates. Hypothesis 2 was supported.

Hypothesis 3 predicted that leader self-serving behavior would mediate the positive relationship between leader greed and employee unethical behavior. The indirect effect of leader greed on employee unethical behavior through leader self-serving behavior was positive and significant (indirect effect = 0.06, 95% Monte Carlo CI = [0.003, 0.143]). Hypothesis 3 was supported.

Table 3 reports the unstandardized results of the multilevel path model.

**TABLE 3:** Unstandardized results of the multilevel path model (Study 1).

	Leader self-serving behavior			Employee unethical behavior		
Variable	Estimate	SE	*p*	Estimate	SE	*p*
Intercept	0.71	0.65	0.276	2.05***	0.26	0.000
Level 1 (Individual)						
Leader self-serving behavior (within)				−0.01	0.20	0.951
Employee age				0.01	0.06	0.828
Employee gender				−0.08	0.06	0.231
Employee education				−0.08	0.06	0.186
Employee work experience				0.05	0.05	0.361
Employee tenure				−0.03	0.04	0.499
Time with leader				−0.04	0.04	0.273
Level 2 (Leader)						
Leader self-serving behavior (between)	0.30**	0.10	0.004	0.21*	0.10	0.032
Leader greed (T1)				−0.05	0.07	0.539
Leader age	0.37*	0.15	0.017	−0.05	0.11	0.653
Leader gender	−0.24	0.19	0.205	−0.28*	0.13	0.039
Leader education	−0.09	0.12	0.457	−0.08	0.08	0.353
Leader work experience	−0.18	0.17	0.304	−0.01	0.12	0.952
Leader tenure	0.04	0.08	0.660	0.09	0.05	0.104
N (Level 1)				231		
N (Level 2)	62			62		
Level-2 variance (τ00)				0.100		
Level-1 residual (σ^2^)				0.168		
*R*^2^/Pseudo-*R*^2^ (L2)	0.219			0.276		
Pseudo-*R*^2^ (L1)				0.030		

Level 1 *N* = 231; Level 2 *N* = 62. The mediator model (M) was estimated at the leader level (*N* = 62) via OLS with leader means; the outcome model (Y) was estimated via multilevel modeling with random intercept at the leader level. Leader greed and Level-2 controls were grand-mean centered; leader self-serving behavior was decomposed into between- and within-leader components.

***p < 0.001, **p < 0.01, *p < 0.05.

Overall, Study 1 provides support for the hypothesized mediation model: trait-level greed in leaders is associated with unethical conduct among their subordinates via leaders’ own enacted self-serving behavior. However, the zero-order correlation between leader self-serving behavior and employee unethical behavior was non-significant (*r* = 0.17, *p* = 0.19), whereas path *b* was statistically significant in the path model (*b* = 0.21, *p* = 0.032); this pattern may indicate a potential suppression effect driven by covariates. To rule out a control-driven suppression effect, we re-estimated the outcome equation (Bernerth and Aguinis, 2016). The results remained consistent without control variables (indirect effect = 0.055, 95% Monte Carlo CI = [0.004, 0.129]).

## 4 Study 2

### 4.1 Methods

#### 4.1.1 Sample and procedure

We designed Study 2 to provide a constructive replication and extension of Study 1 in two important ways. First, to broaden the generalizability of our findings beyond the specific organizational sample in Study 1, we recruited a diverse sample of full-time employees working across a wide range of organizations in China. Second, to address the hypothesized boundary role of individual differences in moral awareness, we introduced employee moral attentiveness (Reynolds, 2008) as a theoretical moderator of the mediated relationship. Participants were not sampled from the same teams or organizations, and no two reported on the same leader. The data therefore contain no clustering structure, and single-level regression with conventional standard errors is appropriate.

Data were collected in three waves, separated by 2 weeks. At Time 1 (T1), participants reported their own trait-level employee moral attentiveness, their leader’s dispositional greed, and demographic information. At Time 2 (T2), participants rated their direct leader’s self-serving behavior. At Time 3 (T3), participants self-reported their own unethical behavior at work. We matched participants’ responses across the three waves using unique participant IDs. After listwise deletion, the final sample consisted of 401 employees (27.9% female; mean age = 32.3 years, *SD* = 5.5).

#### 4.1.2 Measures

Following Brislin’s (1980) back-translation procedure, all items were translated from English into Chinese by two bilingual researchers and back-translated by two independent researchers. Discrepancies were resolved through discussion.

##### 4.1.2.1 Employee moral attentiveness (T1)

We measured employees’ trait-level moral attentiveness with Reynolds’s (2008) 11-item scale. Responses were provided on a five-point Likert-type scale (1 = strongly disagree; 5 = strongly agree). Cronbach’s α = 0.92.

##### 4.1.2.2 Leader greed (T1)

We used the same seven leader greed items as in Study 1 but administered them using a seven-point Likert-type response scale (1 = strongly disagree; 7 = strongly agree) to increase response variability in this larger sample. Cronbach’s α = 0.91.

##### 4.1.2.3 Leader self-serving behavior (T2)

We used the same 8-item scale as in Study 1 (1 = strongly disagree; 7 = strongly agree). Cronbach’s α = 0.91.

##### 4.1.2.4 Employee unethical behavior (T3)

Since the outcome variable in Study 2 concerned the focal employee’s own conduct, we operationalized employee unethical behavior as a self-report measure (1 = strongly disagree; 7 = strongly agree) using the same 5-item scale as in Study 1. Self-report measures have been used in prior research to assess individuals’ own unethical behavior (Kish-Gephart et al., 2010). Cronbach’s α = 0.90.

##### 4.1.2.5 Control variables

Following recommendations regarding the use of control variables (Bernerth and Aguinis, 2016), we controlled for employees’ age, gender, education, organizational tenure, and work experience, and leader gender, all measured at T1.

#### 4.1.3 Analytical strategy

Given that Study 2 utilized a single-level, individual-level design, we tested our hypotheses using ordinary least squares (OLS) regression implemented in a PROCESS-compatible framework (Hayes, 2017). Specifically, we estimated a hierarchical sequence of four regression models predicting employee unethical behavior—Model 1 (total effect of leader greed), Model 2 (adding leader self-serving behavior to test the direct and indirect effects), Model 3 (adding employee moral attentiveness as a main effect), and Model 4 (adding the interaction term between leader self-serving behavior and employee moral attentiveness to test H4)—together with a mediator equation regressing leader self-serving behavior on leader greed (H1). Leader greed, leader self-serving behavior, and employee moral attentiveness were mean-centered prior to computing the interaction term (Aiken et al., 1991).

To test Hypotheses 3 and 5, we employed the Monte Carlo method for assessing mediation with 20,000 simulated draws (Preacher and Selig, 2012; Selig and Preacher, 2008). The Monte Carlo method simulates the joint sampling distribution of the *a* coefficient (from the mediator equation) and the conditional *b* coefficient (*b*_2_ + *b*_4_⋅*W*, from the outcome equation) directly from their asymptotic multivariate-normal distributions, and therefore provides accurate confidence intervals for the product distribution *a* × (*b*_2_ + *b*_4_⋅W) without requiring computationally expensive resampling or relying on the normality of the indirect effect itself. Following Hayes (2015, 2017), we computed the Index of Moderated Mediation (IMM = *a* × *b*_4_) and its 95% Monte Carlo CI to formally test whether the strength of the indirect effect varies as a function of employee moral attentiveness, and we probed the conditional indirect effect at −1 *SD*, the mean, and +1 *SD* of employee moral attentiveness.

### 4.2 Results

#### 4.2.1 Descriptive analysis

Table 4 presents means, standard deviations, Cronbach’s alphas, and zero-order correlations. Consistent with our hypothesized model, leader greed was positively correlated with leader self-serving behavior (*r* = 0.38, *p* < 0.001) and with employee unethical behavior (*r* = 0.39, *p* < 0.001); leader self-serving behavior was positively correlated with employee unethical behavior (*r* = 0.51, *p* < 0.001); and employee moral attentiveness was negatively correlated with all three focal variables (*r*s = −0.64 to −0.14, all *p*s < 0.01), providing initial support for the hypothesized pattern.

**TABLE 4:** Means, standard deviations, reliabilities, and correlations among core study variables (Study 2).

Variables	M	SD	1	2	3	4
1. Leader greed (T1)	3.56	1.06	(0.91)	(0.91)	(0.92)	(0.90)
2. Leader self-serving behavior (T2)	3.49	1.05	0.38***			
3. Employee moral attentiveness (T1)	2.93	0.71	−0.17***	−0.14**		
4. Employee unethical behavior (T3)	3.63	1.16	0.39***	0.51***	−0.64***	

*N* = 401. Cronbach’s alphas are on the diagonal in parentheses.

***p < 0.001, **p < 0.01, T1 = Time 1; T2 = Time 2; T3 = Time 3.

#### 4.2.2 Confirmatory factor analyses

Following common practice (Little et al., 2002; Zhang et al., 2024), we created three balanced item parcels for each latent construct and examined the discriminant validity of employee moral attentiveness, leader greed, leader self-serving behavior, and employee unethical behavior. As reported in Table 5, the hypothesized four-factor CFA model demonstrated excellent fit to the data: χ^2^(48) = 42.37, CFI = 1.00, TLI = 1.00, and RMSEA = 0.000. This model fit significantly better than every plausible alternative: (a) a three-factor model combining leader greed and leader self-serving behavior [Δχ^2^(3) = 729.79, *p* < 0.001]; (b) a three-factor model combining leader self-serving behavior and employee unethical behavior [Δχ^2^(3) = 608.79, *p* < 0.001]; (c) a three-factor model combining employee moral attentiveness and leader greed [Δχ^2^(3) = 823.18, *p* < 0.001]; (d) a two-factor model distinguishing only employee moral attentiveness from the remaining three variables [Δχ^2^(5) = 1250.74, *p* < 0.001]; and (e) a one-factor model [Δχ^2^(6) = 1836.02, *p* < 0.001]. These results support the discriminant validity of our four focal constructs.

**TABLE 5:** Results of confirmatory factor analyses (Study 2).

Model	χ^2^	df	χ^2^/df	CFI	TLI	RMSEA	Δχ^2^
Four-factor model (hypothesized)	42.37	48	0.88	1.000	1.000	0.000	
Three-factor: LG + LSSB combined	772.16	51	15.14	0.801	0.743	0.188	729.79***
Three-factor: LSSB + EUB combined	651.15	51	12.77	0.835	0.786	0.172	608.79***
Three-factor: EMA + LG combined	865.55	51	16.97	0.776	0.710	0.200	823.18***
Two-factor: EMA vs. (LG + LSSB + EUB)	1293.10	53	24.40	0.658	0.574	0.242	1250.74***
One-factor model	1878.38	54	34.78	0.497	0.386	0.291	1836.02***

*N* = 401. LG, leader greed; LSSB, leader self-serving behavior; EUB, employee unethical behavior; EMA, employee moral attentiveness.

***p < 0.001

##### 4.2.2.1 Common method bias

Since all variables in Study 2 were reported by the same source, we evaluated the risk of common method variance (CMV). First, we adopted several procedural remedies recommended by Podsakoff et al. (2003), including a three-wave, time-lagged design with a two-week interval between adjacent waves and the use of different cognitive frames for reporting employee moral attentiveness, leader behavior, and employees’ own conduct. Second, since no marker variable was collected at the design stage, we implemented an unmeasured latent method factor (ULMF) test (Podsakoff et al., 2003; Williams et al., 1989). Specifically, we re-estimated the four-factor measurement model by adding a latent method factor on which all indicators loaded. The method factor was specified as orthogonal to the substantive factors. Adding the method factor did not significantly improve fit, Δχ^2^(12) = 16.95, *p* = 0.151. On average, the method factor accounted for only 2.7% of the indicator variance, whereas the substantive factors accounted for 74.5%. The variance attributed to the method factor was also substantially lower than the average method variance of approximately 25% reported in published research (Williams et al., 1989). Moreover, all substantive factor loadings remained significant. Third, as supplementary evidence, Harman’s single-factor test showed that the first unrotated factor accounted for 32.16% of the total variance (below the conventional 50% threshold), and the single-factor CFA fit the data poorly (CFI = 0.50, RMSEA = 0.29). Taken together, these results suggest that CMV is unlikely to fully account for the observed relationships in Study 2.

### 4.2.3 Hypothesis testing

First, we examined multicollinearity. No standardized coefficient exceeded 1 in any Study 2 model (largest | β| = 0.56, for employee moral attentiveness). Variance inflation factors (VIFs) for all focal predictors were low (leader greed = 1.22; leader self-serving behavior = 1.20; employee moral attentiveness = 1.07; interaction = 1.08). Among control variables, organizational tenure and total work experience were naturally collinear with each other and with age (VIF up to 30.7); a robustness model dropping work experience reduced the maximum VIF to 6.29, and all focal coefficients were unchanged to three decimal places.

Hypothesis 1 predicted that leader greed would be positively associated with leader self-serving behavior. In the mediator equation, after controlling for all demographic covariates, leader greed was a significant positive predictor of leader self-serving behavior (*b* = 0.38, *SE* = 0.05, *p* < 0.001). Hypothesis 1 was supported.

Hypothesis 2 predicted that leader self-serving behavior would be positively associated with employee unethical behavior. In Model 2 of the outcome equation, after controlling for leader greed and all demographic covariates, leader self-serving behavior was a significant positive predictor of employee unethical behavior (*b* = 0.47, *SE* = 0.05, *p* < 0.001). Hypothesis 2 was supported.

Hypothesis 3 predicted that leader self-serving behavior would mediate the positive relationship between leader greed and employee unethical behavior. The Monte Carlo-estimated indirect effect of leader greed on employee unethical behavior through leader self-serving behavior was positive and significant (indirect effect = 0.145, 95% Monte Carlo CI = [0.103, 0.192]). Hypothesis 3 was supported.

Table 6 reports the unstandardized coefficients of the hierarchical regression models, and Table 7 reports the Monte Carlo results for the indirect, conditional indirect, and moderated-mediation effects.

**TABLE 6:** Results of hierarchical regression analyses (Study 2).

	LSSB		Model 1 (EUB)		Model 2 (EUB)		Model 3 (EUB)		Model 4 (EUB)	
Variable	B	SE	B	SE	B	SE	B	SE	B	SE
Constant	2.70	(0.64)	3.78	(0.71)	4.15	(0.64)	3.47	(0.48)	3.06	(0.46)
Gender	0.01	(0.11)	−0.01	(0.12)	−0.01	(0.11)	−0.03	(0.09)	0.01	(0.08)
Age	0.02	(0.02)	−0.01	(0.03)	−0.02	(0.02)	0.01	(0.02)	0.03^†^	(0.02)
Education	0.05	(0.07)	0.04	(0.08)	0.02	(0.07)	0.00	(0.05)	−0.02	(0.05)
Organizational tenure	−0.01	(0.05)	−0.02	(0.06)	−0.02	(0.05)	−0.02	(0.04)	−0.04	(0.04)
Work experience	−0.02	(0.04)	0.02	(0.05)	0.03	(0.04)	0.00	(0.03)	0.01	(0.03)
Leader gender	0.08	(0.10)	−0.04	(0.11)	−0.08	(0.10)	−0.08	(0.08)	−0.02	(0.07)
Leader greed (X)	0.38***	(0.05)	0.43***	(0.05)	0.25***	(0.05)	0.17***	(0.04)	0.19***	(0.04)
Leader self-serving behavior (M)					0.47***	(0.05)	0.41***	(0.04)	0.39***	(0.04)
Employee moral attentiveness (W)							−0.93***	(0.05)	−0.88***	(0.05)
Interaction (M × W)									−0.35***	(0.05)
*R* ^2^	0.156		0.158		0.310		0.611		0.654	

*N* = 401. Unstandardized coefficients are reported, with standard errors in parentheses. Leader self-serving behavior and employee moral attentiveness were mean-centered prior to computing the interaction term. LSSB, leader self-serving behavior; EUB, employee unethical behavior.

***p < 0.001, ^†^*p* < 0.10.

**TABLE 7:** Monte Carlo results for the indirect and moderated indirect effects (Study 2).

Effect	Estimate	SE	95% Monte Carlo CI
Indirect effect (a × b)	0.145	0.023	[0.103, 0.192]
Conditional indirect effects			
At low employee moral attentiveness (−1 SD)	0.236	0.034	[0.171, 0.306]
At high employee moral attentiveness (+1 SD)	0.054	0.021	[0.014, 0.097]
Index of moderated mediation (a × b_4_)	−0.129	0.025	[−0.181, −0.084]

*N* = 401.

Hypothesis 4 predicted that employee moral attentiveness would moderate the relationship between leader self-serving behavior and employee unethical behavior, such that the positive link would be weaker when employee moral attentiveness was high and stronger when employee moral attentiveness was low. In Model 4 of the outcome equation, the interaction between leader self-serving behavior and employee moral attentiveness was significant in the predicted direction (*b*_4_ = −0.35, *SE* = 0.05, *p* < 0.001). To probe the form of the interaction, we computed simple slopes at −1 SD, the mean, and +1 SD of employee moral attentiveness (see Figure 2). The positive relationship between leader self-serving behavior and employee unethical behavior was strongest when employee moral attentiveness was low (slope = 0.63, *SE* = 0.05, *p* < 0.001), and was attenuated when employee moral attentiveness was high (slope = 0.14, *SE* = 0.05, *p* = 0.007). This pattern supports Hypothesis 4.

**FIGURE 2:**
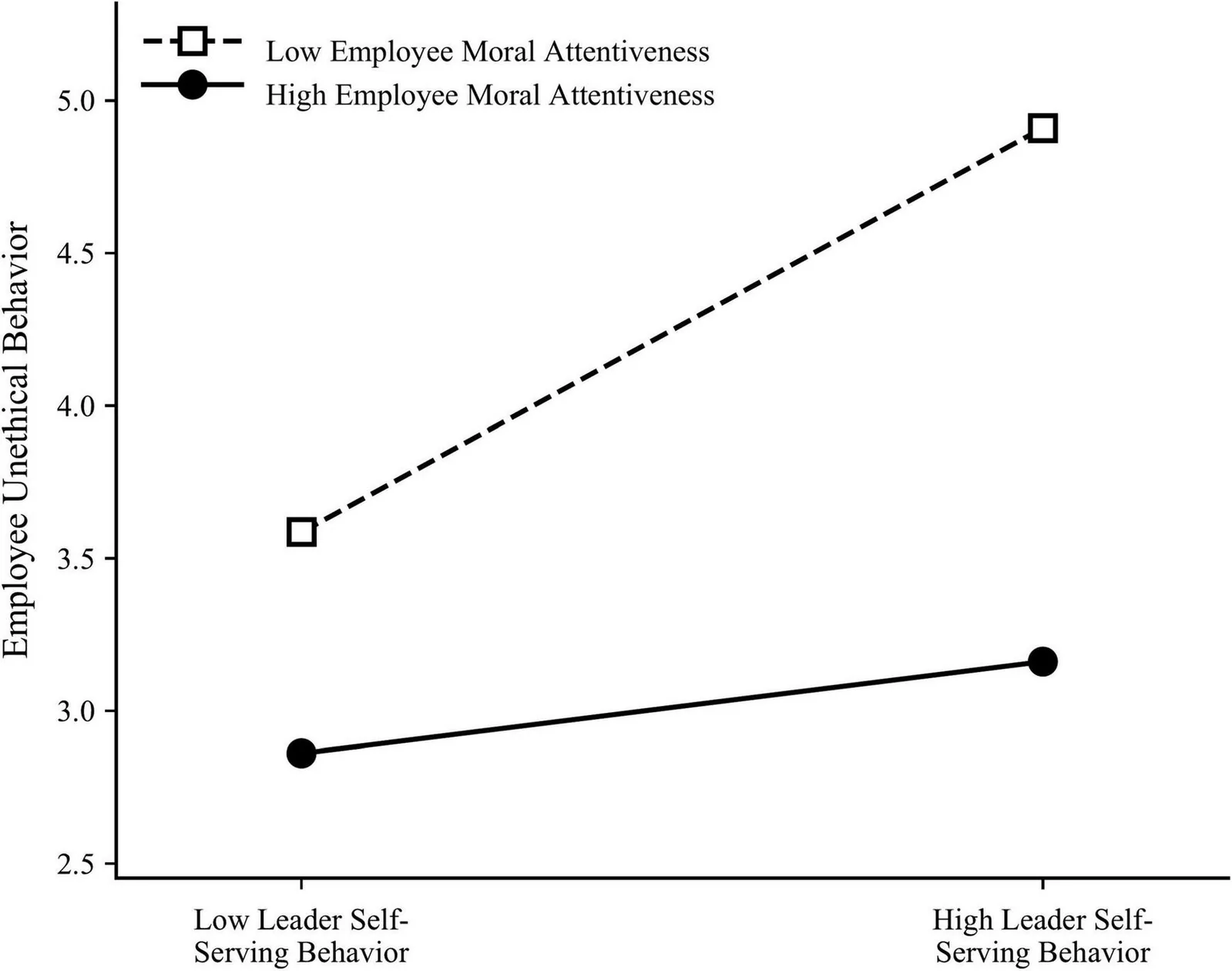
The moderating effect of employee moral attentiveness on the relationship between leader self-serving behavior and employee unethical behavior.

Hypothesis 5 predicted that employee moral attentiveness would moderate the indirect effect of leader greed on employee unethical behavior through leader self-serving behavior. As shown in Table 7, the Index of Moderated Mediation (Hayes, 2015, 2017) was negative and significant (IMM = −0.129, 95% Monte Carlo CI = [−0.181, −0.084]), indicating that the strength of the indirect effect varied with employee moral attentiveness. Consistent with this, the conditional indirect effect was significant at low (*ab* = 0.236, 95% Monte Carlo CI = [0.171, 0.306]), mean (*ab* = 0.145, 95% Monte Carlo CI = [0.103, 0.192]), and high (*ab* = 0.054, 95% Monte Carlo CI = [0.014, 0.097]) levels of employee moral attentiveness. Thus, as employee moral attentiveness rises, the indirect pathway through which leader greed relates to employee unethical behavior is substantially weakened, though it remains significant. Hypothesis 5 was supported.

## 5 Discussion

Drawing on social cognitive theory (Bandura, 1986, 1991) and the trickle-down model (Mayer et al., 2009), we examined the relationships among leader greed, leader self-serving behavior, and employee unethical behavior, as well as the moderating role of employee moral attentiveness. The results indicated that leader greed was positively associated with leader self-serving behavior and that leader self-serving behavior mediated the positive association between leader greed and employee unethical behavior. This pattern is consistent with an observational-learning account in which employees may interpret leader self-serving behavior as signaling that prioritizing personal interests is acceptable (Bandura, 1986; Mayer et al., 2009). When personal interests conflict with ethical standards, such an interpretation may increase the likelihood of unethical behavior (Bandura, 1999). Furthermore, employee moral attentiveness moderated the positive association between leader self-serving behavior and employee unethical behavior, such that the association was stronger at lower levels of moral attentiveness and weaker at higher levels. The corresponding conditional indirect effect linking leader greed to employee unethical behavior through leader self-serving behavior was likewise weaker at higher levels of employee moral attentiveness.

### 5.1 Theoretical implications

First, our study extends greed research beyond its predominantly intrapersonal focus by examining the interpersonal influence of greed, thereby expanding the theoretical boundaries of greed research and enriching our understanding of how leader greed relates to employee ethical behavior. Prior research has largely centered on the outcomes experienced and behaviors exhibited by greedy individuals themselves (Crusius et al., 2021; Bao et al., 2022; Weller et al., 2026). Wang and Murnighan (2011), however, suggested that greed contributes to important personal and interpersonal dynamics, while also noting the relative scarcity of empirical research examining these dynamics. Subsequent conceptual work and review articles have further highlighted the interpersonal and cross-level implications of greed (Carnevale et al., 2021; Takacs-Haynes et al., 2026). By examining the relationship between leader greed and employee unethical behavior, our study provides empirical evidence consistent with this emerging interpersonal perspective and extends greed research to the domain of employee ethical behavior.

Relatedly, this interpersonal contribution situates leader greed within the broader literature on the dark side of leadership. Although dispositional greed is moderately correlated with narcissism (*r* = 0.266, *p* < 0.001) and Machiavellianism (*r* = 0.263, *p* < 0.001), these constructs differ in their defining emphases (Sekhar et al., 2020). Greed centers on always wanting more and never being satisfied with what one currently has (Seuntjens et al., 2015). Narcissism is characterized by a grandiose self-view and the pursuit of admiration (Haynes et al., 2017; Sekhar et al., 2020), whereas Machiavellianism emphasizes a cynical orientation toward morality and strategic manipulation in pursuit of self-interest (Sekhar et al., 2020). By contrast, unethical leadership is defined primarily in terms of leaders’ decisions and behaviors that violate legal or moral standards, as well as the processes and structures through which they encourage unethical behavior among followers (Brown and Mitchell, 2010; Hassan et al., 2023). Taken together, these distinctions suggest that leader greed offers a distinct motivational lens for understanding the dark side of leadership because it is characterized by an insatiable desire to acquire more (Krekels and Pandelaere, 2015; Seuntjens et al., 2015; Zeelenberg and Breugelmans, 2022). In our theoretical model, this defining characteristic helps explain why leader greed may be expressed through leader self-serving behavior and how such behavior may be associated with employee unethical behavior.

Second, we identify leader self-serving behavior as a mediating pathway, thereby clarifying how the interpersonal influence of leader greed may trickle down to employees. Leader greed, as a dispositional tendency, may be expressed in self-serving behavior, such as claiming undue credit for group accomplishments or using a leadership position to secure personal benefits (Rus et al., 2010a,b). Drawing on social cognitive theory (Bandura, 1986) and the trickle-down model (Mayer et al., 2009), our study provides evidence of an indirect association between leader greed and employee unethical behavior through leader self-serving behavior. Because leaders serve as salient behavioral referents whose conduct can signal what is acceptable (Mayer et al., 2009), employees may interpret leader self-serving behavior as indicating that similar conduct is acceptable (Bandura, 1986; Mayer et al., 2009) and perceive leader greed as implicit encouragement of unethical behavior (Zhou et al., 2026). Such interpretations may weaken employees’ internal moral self-restraint and make unethical conduct easier to justify (Bandura, 1999).

Third, we identify employee moral attentiveness (Reynolds, 2008) as an important boundary condition that helps explain why employees may exhibit different levels of unethical behavior when facing leader greed. Social cognitive theory emphasizes that individuals are not passive recipients of environmental signals but actively process and interpret situational cues through their internal cognitive schemas (Bandura, 1991). Moral attentiveness may therefore serve as a perceptual lens that shapes how employees attend to and interpret leader self-serving behavior (Reynolds, 2008). Employees high in moral attentiveness are more likely to perceive and evaluate such behavior through a moral frame (Reynolds, 2008; van Gils et al., 2015), directing greater attention to the behavior’s ethical implications and, in turn, potentially reducing their likelihood of engaging in unethical behavior. In contrast, employees low in moral attentiveness are less likely to perceive and evaluate leader behavior in moral terms and are less sensitive to the moral cues embedded in such behavior (Reynolds, 2008; van Gils et al., 2015). Consequently, they may be more likely to construe such situations as amoral, that is, as falling outside the domain of moral judgment (Tenbrunsel and Smith-Crowe, 2008; van Gils et al., 2015). Under these circumstances, they may be more inclined to interpret leader self-serving behavior through an instrumental frame (Desmet et al., 2015), focusing more on its potential utility than on its ethical implications and thereby strengthening the positive association between such behavior and their own unethical behavior. These findings extend research on moral attentiveness in the ethical leadership literature (van Gils et al., 2015) and help explain how and under what conditions leader greed is associated with employee unethical behavior.

### 5.2 Practical implications

The findings of this study offer several implications for organizational practice.

First, organizations could consider candidates’ dispositional greed when selecting and promoting leaders. Our findings suggest that leader greed may increase the risk of employee unethical behavior. Given that leader greed has also been associated with a range of adverse organizational outcomes (Haynes et al., 2017; Takacs-Haynes et al., 2026), candidates’ integrity and past ethical conduct could be considered alongside performance-based criteria (Mesdaghinia et al., 2023). Specifically, organizations could assess candidates’ past ethical conduct by examining how they have handled resource allocation and conflicts of interest in previous roles.

Second, organizations could strengthen oversight of and accountability for leaders’ use of power. Leaders serve not only as decision-makers but also as important behavioral referents for employees (Mayer et al., 2009). Given that leader self-serving behavior mediated the positive association between leader greed and employee unethical behavior, organizations may benefit from paying closer attention to how managers exercise authority and balance personal and collective interests. Organizations could also reinforce managers’ awareness of role boundaries through relevant training and help them understand how their conduct may be interpreted by subordinates. Furthermore, organizations could review managers’ performance evaluation and compensation arrangements to avoid excessive emphasis on individual gains and to incorporate team-oriented and ethical criteria, paying particular attention to the design and negotiation of executive compensation (Haynes et al., 2017; Sajko et al., 2021).

Third, the buffering role of employee moral attentiveness has implications for ethics training and employee development. Our findings indicate that moral attentiveness weakens the positive association between leader self-serving behavior and employee unethical behavior. Organizations could incorporate regular case-based discussions and related learning activities into employee training programs to help employees recognize and consider the moral dimensions of workplace situations (Reynolds, 2008; Wurthmann, 2013). Such initiatives may encourage employees to attend more closely to the ethical implications of leader behavior and make more deliberate moral judgments in complex organizational contexts.

### 5.3 Limitations and future research

Despite its contributions, this study has several limitations that point to promising avenues for future research. First, both studies have limitations related to research design, measurement, and data sources. In Study 1, the use of matched leader–employee data reduced same-source concerns, but the three-factor CFA yielded less-than-ideal fit indices (TLI = 0.88; RMSEA = 0.105). RMSEA should be interpreted cautiously because it can be inflated in models with small degrees of freedom (Kenny et al., 2015), while comparisons with alternative nested models nevertheless supported the distinctiveness of the three focal constructs. The leader-rated measure of employee unethical behavior also demonstrated modest reliability (α = 0.66; ω = 0.65), which may have reduced estimate precision and attenuated associations involving this measure. Future research should seek to replicate these findings using a measure with stronger psychometric properties. In Study 2, all focal variables were assessed using employee self-reports. Although the three-wave temporal separation, the ULMF results (average method variance = 2.7%), and the poor fit of the single-factor model collectively suggest that common method variance is unlikely to fully account for the findings, self-reports of one’s own unethical behavior remain susceptible to social desirability bias. The multisource design of Study 1, in which leaders rated employee unethical behavior, provides a useful complement to Study 2 by reducing reliance on same-source self-reports. Future research could incorporate a marker variable and supplement self-reports with behavioral or other-source assessments of employee unethical behavior. Moreover, although both studies employed three-wave, time-lagged designs, their nonexperimental nature does not permit causal inferences. Future research could employ scenario-based experiments that manipulate the level of leader greed, thereby permitting stronger causal inferences about its effects on employee behavior and work outcomes.

Second, regarding underlying mechanisms and boundary conditions, our study focused on leader self-serving behavior as a mediating pathway and employee moral attentiveness as a boundary condition. However, other psychological and relational mechanisms may also help explain how leader greed is associated with employee outcomes and therefore warrant examination through alternative theoretical lenses. For example, from a psychological contract perspective (Rousseau, 1989), leader greed and associated leader self-serving behavior may be perceived by employees as breaching their psychological contracts. Such perceived breach may, in turn, lead employees to feel unfairly treated and motivate them to engage in equity-restoring responses (Kickul and Lester, 2001). Alternatively, from a social exchange perspective (Cropanzano and Mitchell, 2005), leader self-serving behavior may signal a disregard for employees’ interests and elicit negative reciprocity (Gouldner, 1960). Employees may consequently reciprocate by engaging in organizational deviance (Mayer et al., 2009). Future research could draw on these perspectives to examine other mediating mechanisms, including employees’ perceptions of distributive justice (building on Camps et al., 2012), perceptions of abusive supervision (Tepper, 2000), and emotional exhaustion (Maslach and Jackson, 1981). Examining these mechanisms could provide a more comprehensive account of how leader greed is associated with employee outcomes. In terms of boundary conditions, future research could also investigate the influence of other individual differences and contextual factors. At the individual level, equity sensitivity may serve as a moderator, as it affects employees’ perceptions of equity and their attitudes and reactions to unfair outcomes (Kickul and Lester, 2001). Additionally, employees’ limited time perspective (Cozzolino et al., 2009) and power distance orientation (Lian et al., 2012) may influence their judgments of and behavioral responses to leader self-serving behavior. At the organizational and team levels, future research could examine ethical climate (Decoster et al., 2021) and team cohesion (Narayanan et al., 2006), respectively, as additional contextual factors, thereby further advancing theorizing about leader greed and its organizational implications.

Third, both studies drew on samples recruited in China, which may limit the generalizability of our findings to other cultural contexts. Cultural value orientations may shape employees’ expectations of leaders as well as their perceptions of and responses to leader behavior (Lian et al., 2012). Accordingly, employees across cultural settings may interpret and respond to leader greed and leader self-serving behavior differently, and the moderating role of moral attentiveness may likewise vary by culture. Future research should replicate the proposed model using samples from diverse cultural settings and employ cross-cultural comparative designs to examine whether the observed relationships and conditional patterns vary across contexts.

## Data Availability

The raw data supporting the conclusions of this article will be made available by the authors, without undue reservation.
